# Racial/Ethnic Disparities in Physical Function Before and After Total Knee Arthroplasty Among Women in the United States

**DOI:** 10.1001/jamanetworkopen.2020.4937

**Published:** 2020-05-15

**Authors:** Alyson M. Cavanaugh, Mitchell J. Rauh, Caroline A. Thompson, John Alcaraz, William M. Mihalko, Chloe E. Bird, Giselle Corbie-Smith, Milagros C. Rosal, Wenjun Li, Aladdin H. Shadyab, Todd Gilmer, Andrea Z. LaCroix

**Affiliations:** 1 Joint Doctoral Program in Public Health, San Diego State University/University of California, San Diego, San Diego; 2 Doctor of Physical Therapy Program, San Diego State University, San Diego, California; 3 Graduate School of Public Health, San Diego State University, San Diego, California; 4 Campbell Clinic Department of Orthopaedic Surgery and Biomedical Engineering, University of Tennessee Health Science Center, Memphis; 5 Health Care Division, RAND, Santa Monica, California; 6 Center for Health Equity Research, University of North Carolina School of Medicine, Chapel Hill; 7 Department of Population and Quantitative Sciences, University of Massachusetts Medical School, Worchester; 8 Department of Medicine, University of Massachusetts Medical School, Worcester; 9 Department of Family Medicine and Public Health, University of California, San Diego, La Jolla

## Abstract

**Importance:**

Although racial/ethnic differences in functional outcomes after total knee arthroplasty (TKA) exist, whether such differences are associated with differences in presurgical physical function (PF) has not been thoroughly investigated.

**Objective:**

To examine trajectories of PF by race/ethnicity before and after TKA among older women.

**Design, Setting, and Participants:**

This cohort study was conducted among the prospective Women’s Health Initiative with linked Medicare claims data. A total of 10 325 community-dwelling women throughout the United States with Medicare fee-for-service underwent primary TKA between October 1, 1993, and December 31, 2014, and were followed up through March 31, 2017.

**Exposures:**

Race/ethnicity comparisons between Hispanic or Latina women, non-Hispanic black or African American women, and non-Hispanic white women (hereafter referred to as Hispanic, black, and white women, respectively).

**Main Outcomes and Measures:**

Physical functioning scale scores and self-reported activity limitations with walking 1 block, walking several blocks, and climbing 1 flight of stairs were measured by the RAND 36-Item Health Survey during the decade before and after TKA, with a median of 9 PF measurements collected per participant over time.

**Results:**

In total, 9528 white women (mean [SD] age at surgery, 74.6 [5.5] years), 622 black women (mean [SD] age at surgery, 73.1 [5.3] years), and 175 Hispanic women (mean [SD] age at surgery, 73.1 [5.2] years) underwent TKA. During the decade prior to TKA, black women had lower PF scores than white women (mean difference, −5.8 [95% CI, −8.0 to −3.6]) and higher odds of experiencing difficulty walking a single block (5 years before TKA: odds ratio, 1.86 [95% CI, 1.57-2.21]), walking multiple blocks (odds ratio, 2.14 [95% CI, 1.83-2.50]), and climbing 1 flight of stairs (odds ratio, 1.81 [95% CI, 1.55-2.12]). After TKA, black women continued to have lower PF scores throughout the decade (mean difference 1 year after TKA, −7.8 [95% CI, −10.8 to −4.9]). After adjusting for preoperative PF scores, PF scores after TKA were attenuated (mean difference 1 year after TKA, −3.0 [95% CI, −5.3 to −0.7]), with no statistically significant differences in long-term follow-up. Hispanic women had similar PF scores to white women during the pre-TKA and post-TKA periods.

**Conclusions and Relevance:**

This study suggests that black women had significantly poorer PF than white women during the decades before and after TKA. Poorer PF after surgery was associated with poorer preoperative PF. Reducing disparities in post-TKA functional outcomes should target maintenance of function preoperatively in the early stages of arthritic disease and/or reduction of delays to receiving TKA once need arises.

## Key Points

**Question:**

Is preoperative physical function associated with racial/ethnic disparities in functional outcomes after total knee arthroplasty among women?

**Findings:**

In this cohort study of 10 325 women who underwent total knee arthroplasty, poorer preoperative physical function among black women was associated with poorer outcomes after total knee arthroplasty.

**Meaning:**

Efforts to reduce disparities in functional outcomes after total knee arthroplasty should be focused preoperatively on optimizing the physical function of black women in the early stages of arthritis and reducing delays to surgical treatment once clinical need arises.

## Teaser

This cohort study uses data from the Women’s Health Initiative to examine trajectories of physical function by race/ethnicity before and after total knee arthroplasty among community-dwelling older women in the United States.

## Introduction

Racial/ethnic disparities in total knee arthroplasty (TKA) are well documented.^1,2,3^ Less studied, but equally important, are the racial/ethnic disparities in outcomes after TKA.^4^ Current evidence suggests that racial/ethnic minority groups experience lower satisfaction, higher postoperative pain, more residual joint stiffness, and poorer physical function (PF) after joint arthroplasty.^4^

Preoperative PF is associated with function after TKA.^5,6^ Limited evidence is currently available regarding the association of preoperative PF and racial disparities in functional outcomes. However, poorer PF among black and Hispanic patients undergoing TKA have been reported.^7,8,9,10^ Little is known regarding the onset and progression of functional limitations prior to TKA. A longer duration of living with limited mobility could be associated with further deterioration in muscle strength, restrictions in joint range of motion, and altered pain pathways.^11,12,13^ Thus, both the level of PF at the time of surgery and the duration of PF limitations may be important factors associated with functional outcomes after TKA. The purpose of this study was to use prospectively collected data from a large cohort of community-dwelling older women to assess functional status trajectories by race/ethnicity during the decades leading to and following TKA.

## Methods

### Participants

This prospective cohort study used data from the Women’s Health Initiative (WHI), which included 2 major parts: clinical trials and an observational study. The design of the WHI is described in detail elsewhere.^14,15^ In brief, the WHI enrolled 161 808 postmenopausal community-dwelling women at 40 US clinical centers between October 1, 1993, and December 31, 1998. Women were followed up until September 2005, and surviving women were then invited to participate in subsequent extension studies from 2005 to the present. Written informed consent was obtained from each participant. Procedures were approved by the institutional review boards at all participating institutions. This study followed the Strengthening the Reporting of Observational Studies in Epidemiology (STROBE) reporting guideline.

**Figure 1.**
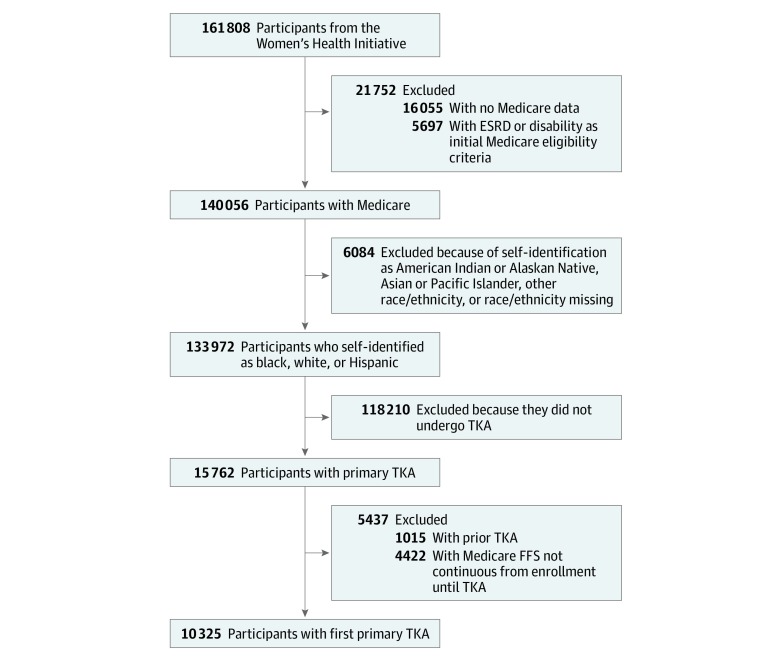
Study Inclusion Flowchart ESRD indicates end-stage renal disease; FFS, fee for service; and TKA, total knee arthroplasty.

Medicare fee-for-service (FFS) claims data have been linked to 145 753 consenting WHI participants. Those who self-identified as non-Hispanic white, non-Hispanic black, or Hispanic or Latina and had Medicare data available were included in this study (Figure 1). Medicare Part A FFS claims were used to identify women who underwent a first TKA from the time of WHI enrollment through December 31, 2014, using the *International Classification of Diseases, Ninth Revision* primary procedure code 81.54. Because the focus was on PF trajectories from time of first TKA, women with prior TKA were excluded. Self-report of joint replacement, other than hip replacement, or a documented procedure code of 81.54 in FFS claims prior to WHI enrollment were used to identify prior TKA. Women without continuous FFS coverage from the time of WHI enrollment or the time of Medicare enrollment until the time of TKA were excluded for lack of surgical history information.

### Measures

The RAND 36-Item Health Survey (RAND-36) PF scale was used to measure PF.^16^ This composite score (range, 0-100, where 0 indicates maximal disability and 100 indicates no limitations) includes 10 survey questions regarding self-perceived difficulty in specific functional activities. Women’s Health Initiative clinical trial participants completed this questionnaire at the screening visit, at 1-year follow-up year 1, and at the end of study, with a 25% subsample completing additional surveys at 3, 6, and 9 years. Surveys were administered to observational study participants at baseline and year 3. Surveys were administered annually to all extension study participants beginning in 2005, and this study included measurements collected through 2017.

Three items of the RAND-36 questionnaire were also analyzed as binary outcomes to determine specific activity limitations prior to TKA. If a woman responded that her health limited her either “a little” or “a lot” in climbing 1 flight of stairs, walking 1 block, or walking several blocks, she was classified as having an activity limitation with the corresponding functional task.

Demographic information was collected through questionnaires administered at the WHI baseline. Race/ethnicity was categorized according to self-identified racial or ethnic group and was limited to the responses of non-Hispanic white, non-Hispanic black or African American, or Hispanic or Latina (referred to hereafter as white, black, and Hispanic, respectively). Self-reported family annual income at baseline (classified as <$20 000, $20 000 to <$50 000, and ≥$50 000) and highest educational level (classified as less than a high school diploma or General Educational Development certificate, high school diploma or General Educational Development certificate, some college or associate degree, and Baccalaureate degree or higher) were used to assess socioeconomic status (SES). Neighborhood SES (NSES), a computed variable based on 2000 census tract-level data on poverty, educational level, and other SES variables within the geographical region, was also included as a measure of SES.^17,18^ Neighborhood SES values range from 0 to 100, with higher values representing more affluent census tracts.

Body mass index (BMI) was measured at baseline using height and weight collected by trained assessors using a wall-mounted stadiometer to the nearest 0.1 cm and a balance beam scale to the nearest 0.1 kg. Body mass index was calculated as weight in kilograms divided by height in meters squared and categorized according to World Health Organization cutpoints (underweight, <18.5; healthy weight, 18.5 to <25; overweight, 25 to <30; obese I, 30 to <35; obese II, 35 to <40; obese III, ≥40).^19^

Marital status, living arrangement, depressive symptoms, and physical activity were determined using the data collection point prior to and most recent to TKA. Marital status was dichotomized (yes or no) according to the response of being currently married or in an intimate relationship. Living arrangement was dichotomized (yes or no) based on whether the participant lived alone. Presence of depressive symptoms was determined using the 8-item scale for depressive disorders by Burnam et al,^20^ which combines 6 questions from the short form of the Center for Epidemiological Studies Depression Scale and 2 items from the Diagnostic Interview Schedule, with a score range of 0 to 1 (where 0 indicates lowest probability of having a depressive disorder and 1 indicates highest probability). A cutoff of 0.06 was used to determine the presence of depressive symptoms.^21^ Time spent in moderate to vigorous physical activity was based on self-reported frequency and duration of activities of various intensities. Moderate to vigorous physical activity was classified as greater than or equal to 150 minutes per week, 1 minute per week to less than 150 minutes per week, or none.

Medicare FFS claims were used to determine age at TKA and multimorbidity scores. Multimorbidity scores were calculated using the Centers for Medicare & Medicaid Services Hierarchical Conditions Categories risk adjustment, with scores lower than 1.0 indicating relatively healthier individuals. Medicare claims from hospital inpatient and outpatient treatment and physician claims starting 1 year prior to the date of surgery were included to calculate dichotomous values for the 70 Hierarchical Conditions Categories conditions included in the Centers for Medicare & Medicaid Services Hierarchical Conditions Categories risk score.^22,23^

### Statistical Analysis

#### Assessment of Pre-TKA Functional Status and Temporal Changes

Physical function 1 year prior to surgery for each participant was estimated using generalized linear mixed modeling. Physical function scores from the 10-year period prior to date of surgery were included in the modeling to estimate PF 1 year prior to surgery. With the use of multiple imputations, missing PF measurements for intervals of less than 1 year, 1 to less than 2 years, 2 to less than 5 years, and 5 to 10 years prior to TKA were imputed. Multiple imputation using chained equations was performed with variables of PF, age, time of PF collection, quadratic time, and cubic time and auxiliary variables of baseline PF, time from baseline PF to TKA, and multimorbidity. Twenty-five imputed data sets were created. Generalized linear mixed modeling was then performed using the imputed data sets. Unstructured covariance was used to obtain robust estimates, after comparing Akaike information criterion statistics of models fit with unstructured, autoregressive, and Toeplitz structures. Generalized linear mixed modeling included age, a cubic spline with 5 knots for time, and random intercepts to account for differences in PF measurements at the individual level. Results were averaged among the imputed data sets,^24^ and individual PF measurements 1 year prior to TKA was used as a variable for subsequent modeling.

Physical function trajectories over time by race/ethnicity were modeled using generalized estimating equations (GEEs) in the preoperative and postoperative periods. Preoperative PF was modeled for a period of 10 years prior to surgery. Race/ethnicity and age at TKA were independent variables in the GEE models. Quadratic and cubic time variables were included to allow for nonlinear changes in PF over time. Interaction terms for time and race/ethnicity were included to allow for differing rates of change in PF by race/ethnicity. Interactions were retained in the model if the Wald test statistic was significant at *P* < .10. Because differences in PF between racial/ethnic groups before and after TKA would be compared, women without both pre-TKA and post-TKA PF measurements were excluded from the PF GEE models. To examine whether race/ethnicity associations with preoperative PF varied by SES, the variables NSES, income, and educational level were stratified at their median values, and interaction terms for each were added into 3 separate GEE models. An autoregressive working correlation structure was used for all GEE models.

Activity limitations by race/ethnicity in the decade before TKA were assessed using 3 GEE models with binary outcomes of difficulty walking 1 block, difficulty walking several blocks, and difficulty climbing 1 flight of stairs. Race/ethnicity, time, and interactions of race/ethnicity and time were included using the same method as described for the PF GEE models. Estimated probabilities of experiencing difficulties in the 3 activity limitations were determined during a 10-year interval prior to surgery.

#### Assessment of Post-TKA Functional Status and Temporal Changes

Unadjusted mean values of PF both before and after TKA were calculated at annual increments for each racial/ethnic group and included all available PF scores collected within 364 days of the start of the year. For example, year 1 after TKA included PF scores collected at day 365 through day 729. Scores collected during the first postoperative year were not included because of an assumed temporary decline during the initial postoperative recovery period.^25^

Generalized estimating equation modeling in the postoperative decade used PF measurements collected from 365 days after TKA through year 10 and applied the same method as described for the pre-TKA period. To evaluate whether preoperative PF was associated with racial/ethnic differences in postoperative PF, participants’ estimated 1-year preoperative PF scores as determined by generalized linear mixed modeling were included as an independent variable in the postoperative PF GEE model. Specific time periods of 1 and 2 years, 5 years, and 10 years from TKA were selected to examine disparities in short-term, midterm, and long-term outcomes, respectively.

Because of the large number of women without post-TKA PF measures, a sensitivity analysis using censoring weighting was performed. The inverse of the estimated probability of remaining in the study^26^ (having available post-TKA PF measures) was used to determine individual weights for pre-TKA and post-TKA PF GEE models. Probabilities of remaining in the study were estimated by logistic regression using factors of baseline PF and BMI, 1-year pre-TKA PF, age at TKA, race/ethnicity, multimorbidity, depression, moderate to vigorous physical activity, income, educational level, NSES, and region. Confidence intervals were determined based on a bootstrap distribution with 500 resampling iterations.

All *P* values were from 2-sided tests and results were deemed statistically significant at *P* < .05. All analyses were performed using SAS, version 9.4 (SAS Institute Inc).

## Results

Overall, 10 325 women from the WHI who underwent primary TKA were included, with 9528 (92.3%) self-identifying as white, 622 (6.0%) as black, and 175 (1.7%) as Hispanic (Table 1). The mean (SD) age at TKA was 74.6 (5.5) years for white women, 73.1 (5.2) years for Hispanic women, and 73.1 (5.3) years for black women. Compared with white women, black and Hispanic women were less likely to be married, had lower income, lower educational attainment, lower NSES, higher BMI, and lower participation in moderate to vigorous physical activity. Black women had higher multimorbidity scores, whereas Hispanic women had lower multimorbidity scores, compared with white women.

**Table 1.** Demographic and Health Characteristics of Study Participants by Race/Ethnicity

Characteristic	Participants, No. (%)^a^		
	White	Black	Hispanic or Latina
Age at surgery, mean (SD), y	74.6 (5.5)	73.1 (5.3)	73.1 (5.2)
Region			
Midwest	2085 (21.9)	144 (23.2)	22 (15.8)
Northeast	2671 (28.0)	98 (15.8)	90 (51.4)
South	3070 (32.2)	335 (53.9)	54 (30.9)
West	1702 (17.9)	45 (7.2)	
Married, yes	6519 (68.8)	335 (54.1)	112 (65.1)
Live alone, yes	2628 (29.8)	200 (35.8)	36 (24.7)
BMI			
Underweight or normal weight (<25)	1815 (19.2)	31 (5.0)	
Overweight (25 to <30)	3323 (35.2)	167 (27.1)	48 (27.8)
Obese			
I (30 to <35)	2473 (26.2)	198 (32.1)	58 (33.5)
II or III (≥35)	1831 (19.4)	220 (35.7)	48 (27.8)
Multimorbidity score (CMS-HCC), median (IQR)	0.61 (0.35-0.92)	0.65 (0.41-0.99)	0.55 (0.35-0.96)
Moderate to strenuous physical activity, min/wk			
None	3786 (39.8)	295 (47.6)	90 (52.0)
1 to <150	3147 (33.1)	209 (33.7)	42 (24.3)
>150	2578 (27.1)	116 (18.7)	41 (23.7)
Depressive symptoms, yes	897 (9.4)	74 (12.0)	35 (20.6)
Educational level			
<High school	248 (2.6)	52 (8.4)	28 (16.2)
High school	1698 (17.9)	89 (14.4)	31 (17.9)
Some college	3569 (37.6)	225 (36.3)	70 (40.5)
College graduate	3977 (41.9)	254 (41.0)	44 (25.4)
Family income, $			
<20 000	939 (10.5)	126 (21.6)	40 (24.7)
20 000 to <50 000	4136 (46.1)	262 (44.9)	69 (42.6)
≥50 000	3897 (43.4)	195 (33.5)	53 (32.7)
Neighborhood SES, mean (SD)	77.3 (7.0)	65.8 (11.4)	70.2 (10.5)

Abbreviations: BMI, body mass index (calculated as weight in kilograms divided by height in meters squared); CMS-HCC, Centers for Medicare & Medicaid Services Hierarchical Conditions Categories; IQR, interquartile range; SES, socioeconomic status.

^a^ Presentation of data is restricted to cells with counts greater than 20.

### PF Before TKA

**Figure 2.**
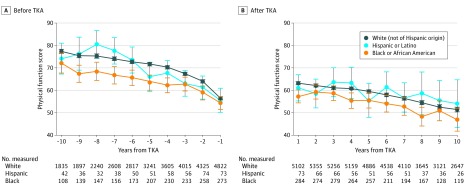
Unadjusted Mean Physical Function Before and After Total Knee Arthroplasty (TKA) by Race/Ethnicity Error bars indicate 95% CIs.

All racial/ethnic groups had declining PF during the decade before TKA, with some functional improvements after TKA (Figure 2). Black women had significantly lower PF scores than white women during the 10-year preoperative period after adjusting for age (mean difference, −5.8 [95% CI, −8.0 to –3.6]). Hispanic women also had slightly lower preoperative PF scores than white women, but this difference was not statistically significant. The odds of experiencing specific activity limitations (walking 1 block, walking several blocks, or climbing 1 flight of stairs) increased in the years approaching surgery for all racial/ethnic groups (eFigure 1 in the Supplement). Although black women had higher odds of experiencing activity limitations during the entire preoperative period compared with white women (difficulty walking a single block at 5 years prior to TKA: odds ratio [OR], 1.86 [95% CI, 1.57-2.21]; difficulty walking multiple blocks: OR, 2.14 [95% CI, 1.83-2.50]; difficulty climbing a flight of stairs: OR, 1.81 [95% CI, 1.55-2.12]), white women experienced higher rates of developing limitations in walking several blocks and climbing stairs in the immediate years preceding TKA compared with black women (Table 2; eFigure 1 in the Supplement). Hispanic and white women were similarly likely to experience walking limitations, but Hispanic women had 67% higher odds of difficulty with climbing a flight of stairs during the year prior to TKA compared with white women (OR, 1.67 [95% CI, 1.21-2.31]).

**Table 2.** Estimated Mean Physical Function Scores and Odds of Activity Limitations at Selected Points Prior to TKA, Women’s Health Initiative, 1993-2017

Time prior to TKA	Physical function, estimated mean (95% CI)^a^	Specific activity limitations, AOR (95% CI)^b^		
		Walking		Climbing a flight of stairs
		1 Block	Several blocks	
1 Year				
Black	53.6 (51.5-55.8)	1.86 (1.57-2.21)	1.91 (1.60-2.28)	1.55 (1.29-1.85)
Hispanic	56.7 (53.2-60.2)	1.06 (0.75-1.49)	1.29 (0.92-1.80)	1.67 (1.21-2.31)
White	59.5 (58.9-60.0)	1 [Reference]	1 [Reference]	1 [Reference]
2 Years				
Black	59.1 (56.9-61.2)	1.86 (1.57-2.21)	1.96 (1.67-2.31)	1.61 (1.36-1.90)
Hispanic	62.2 (58.7-65.6)	1.06 (0.75-1.49)	1.27 (0.94-1.73)	1.66 (1.23-2.23)
White	64.9 (64.3-65.5)	1 [Reference]	1 [Reference]	1 [Reference]
5 Years				
Black	67.1 (65.0-69.3)	1.86 (1.57-2.21)	2.14 (1.83-2.50)	1.81 (1.55-2.12)
Hispanic	70.2 (66.7-73.7)	1.06 (0.75-1.49)	1.22 (0.90-1.66)	1.61 (1.20-2.17)
White	72.9 (72.4-73.5)	1 [Reference]	1 [Reference]	1 [Reference]
10 Years				
Black	73.9 (71.7-76.1)	1.86 (1.57-2.21)	2.48 (1.94-3.17)	2.20 (1.72-2.82)
Hispanic	77.0 (73.5-80.5)	1.06 (0.75-1.49)	1.15 (0.68-1.93)	1.55 (0.96-2.49)
White	79.7 (79.0-80.4)	1 [Reference]	1 [Reference]	1 [Reference]

Abbreviations: AOR, adjusted odds ratio; TKA, total knee arthroplasty.

^a^ Physical function scores correspond to RAND 36-Item Health Survey physical functioning scale. Mean physical function scores estimated through general estimating equations that included the following covariates: age at TKA and linear, quadratic, and cubic time. Mean values correspond to model fit for an age of 74 years at TKA.

^b^ Activity limitations correspond to self-reported difficulty in performing the specific item of the RAND 36-Item Health Survey. Adjusted odds ratios determined through general estimating equations that included age at TKA and linear, quadratic, and cubic time. Time by race/ethnicity interaction term additionally included in models for walking several blocks and climbing a flight of stairs.

Differences in pre-TKA PF between black and white women were more pronounced among women with SES characteristics below median levels compared with those with higher SES levels (eFigure 2 in the Supplement).

### PF After TKA

**Figure 3.**
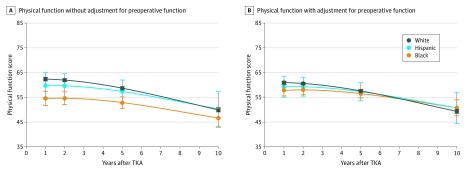
Physical Function in the Decade After Total Knee Arthroplasty (TKA) Without or With Adjustment for Preoperative Function A, Physical function estimates from model fit for age 74 years at time of TKA, adjusted for age. B, Physical function estimates from model fit for age 74 years at TKA and preoperative RAND 36-Item Health Survey physical functioning scale score of 60, adjusted for age and preoperative physical function at 1 year prior to TKA. Error bars indicate 95% CIs.

Black women had significantly lower PF scores during the decade after TKA compared with white women (Figure 3; eTable 1 in the Supplement). At 1 year after TKA, black women had age-adjusted mean PF scores 7.8 points lower than white women (95% CI, −10.8 to −4.9), representing a widening of the gap noted preoperatively (1-year pre-TKA mean difference, −5.8 [95% CI, −8.0 to −3.6]). However, this racial difference in post-TKA PF narrowed over time (10-year post-TKA mean difference, −3.3 [95% CI, −7.1 to 0.4]) because black women, on average, experienced a more gradual decline in function compared with white women (race/ethnicity by time interaction *P* = .06). Hispanic women showed similar mean PF scores compared with white women during the 10-year postoperative period.

Adjustment for 1-year preoperative PF attenuated the racial/ethnic differences in postoperative PF (Figure 3; eTable 1 in the Supplement). After adjustment for preoperative PF, black women still had significantly lower PF at 1 and 2 years after TKA (1-year post-TKA mean difference, −3.0 [95% CI, −5.3 to −0.7]; 2-year post-TKA mean difference, −2.5 [95% CI, −4.5 to −0.4]), but no significant differences persisted between any racial/ethnic groups after year 2. After adjustment for pre-TKA function, black women had a significantly slower overall rate of decline in PF than white women during the decade after TKA.

### Sensitivity Analyses

Women with characteristics of poorer health (higher BMI, multimorbidity, and depression) were more likely to have missing post-TKA PF scores, and there was a statistically significant difference in censoring by race/ethnicity (eTable 2 in the Supplement). The inverse probability of censoring weighting resulted in a strengthening of the association between PF and race/ethnicity during the post-TKA decade.

## Discussion

In a population of community-dwelling older women who underwent TKA, black women had significantly poorer PF scores than white women during the decade before TKA, which persisted during the decade after TKA. After adjustment for preoperative PF, black women had lower PF scores than their white counterparts for the first 2 years after surgery but then had similar PF scores during the remaining years of follow-up. Hispanic and white women had similar preoperative and postoperative PF scores.

The TKA procedure is considered an effective treatment for adults with end-stage knee arthritis and is associated with reduced pain and improved function. However, our findings are consistent with the growing body of evidence that suggests that older black adults do not have the same post-TKA functional outcomes as older white adults.^4^ Although Hispanic ethnicity has been less studied with regard to TKA, our results are congruent with 2 other reports that Hispanic ethnicity was not significantly associated with PF outcomes.^7,27^ Several studies investigating the potential causes of racial/ethnic disparities in post-TKA outcomes have focused on minority groups’ disproportionately higher use of low-volume surgeons or institutions^28,29^ and higher rates of perioperative and postoperative complications, including the development of arthrofibrosis.^30,31,32^ Others have focused on racial/ethnic differences in rehabilitation pathways or postoperative care.^33,34^ However, while preoperative PF is known to be associated with postoperative PF,^5,6^ to date, only few reports have addressed how preoperative PF may be associated with these functional outcome disparities. Kamath et al^35^ performed a retrospective analysis of patients undergoing TKA and reported that African American patients had significantly lower PF scores 2 years after TKA, which was associated with patient self-report of longer delays from onset of symptoms to presentation at the orthopedist office. Lavernia et al^7^ reported that non-Hispanic African American patients had worse functional scores postoperatively than white patients, even after adjusting for worse preoperative functional scores. Both studies used data from surgical institutions where pre-TKA PF and symptom reporting were measured at the patient’s first orthopedist office visit.^7,35^ Our findings, using a community-dwelling cohort, add important and novel information by illustrating the severity of activity limitations and PF impairment, which was present earlier and for a longer duration among black women. Although a gap in function persisted in short-term follow-up, even after adjustment for preoperative function, it is promising to recognize that mid-term and long-term outcomes were similar between race/ethnicity groups after accounting for differences in preoperative PF.

The longer duration of PF impairment among black women is of concern because of the cyclic association with reduction in physical activity, weight gain, and further restrictions in mobility,^36^ all of which are associated with poorer outcomes after TKA.^5^ In addition to poorer PF, black women in this study had higher BMI and lower physical activity before TKA. Although we were unable to measure temporal changes in weight and physical activity prior to TKA because of the timing of data collection for these measures, previous WHI studies have reported that higher BMI and lower physical activity measured at baseline were associated with late-life mobility limitations after TKA, independent of race/ethnicity.^37,38^ Preserving mobility and preventing further functional decline among black women may ultimately need to include strategies such as weight management, promotion of physical activity, or targeted interventions aimed at functional mobility, such as physical therapy. Uptake of evidence-based treatments for arthritis may help slow declines in mobility due to painful joints. However, black adults with arthritis have been shown to be less likely to receive medical or pharmaceutical treatments for pain^39,40^ and are less likely to receive physical therapy services.^41^ Ultimately, identifying black adults with arthritis earlier in the disease process and increasing referrals for and uptake of evidence-based treatments aimed at preserving functional mobility may help reduce the observed disparities both before and after TKA.

Reducing delays to surgery, once the clinical need for TKA arises and conservative treatments become ineffective, may help improve the prognosis postoperatively. In our study, white women who underwent TKA were more likely to develop mobility limitations in the years immediately preceding TKA, whereas black women lived with these mobility limitations several years longer. Similarly, Kamath et al^35^ reported that black women who underwent TKA reported, on average, an additional 20 months of delay from time of onset of disability until time of surgical consultation compared with other racial/ethnic groups. The reasons for delayed surgical intervention likely include differences in health care seeking, referral patterns, and medical access. Although all women in this study had Medicare insurance, fewer economic resources to cover copayments, deductibles, and lost wages may be associated with postponing medical care and/or surgical intervention.^42,43,44^ In our study, women with fewer financial resources experienced poorer PF during the decade prior to TKA, yet the racial/ethnic disparities in preoperative PF were wider in this group than among women with greater SES resources. Mistrust of medical professionals and misconceptions about the benefits and risks of TKA are prevalent among older black adults and have been associated with the underuse of TKA among this group.^45,46,47^ These same factors may play a role in deferral and thus delayed receipt of TKA. In addition, differences between racial/ethnic groups in social responsibilities and resources could be associated with decisions to undergo TKA. Black women were less likely to be married and more likely to live alone; thus, postsurgical care concerns may have led to an initial hesitation to undergo TKA. In addition to economic, cultural, and social factors that may be associated with the timeliness of patients’ decisions to undergo TKA, clinician factors including implicit bias may be associated with referrals, recommendations, and timeliness of surgical treatment. Emerging research suggests that, despite the presence of clinicians’ implicit biases, race/ethnicity does not appear to be associated with physicians’ recommendations for TKA.^48,49^ However, a more thorough understanding of the association of clinician biases with recommendations, patient-clinician communication, and referrals and processes of care from primary to specialist care may elucidate why patients of minority race/ethnicity present at the time of surgery with more advanced disability.^7,50^ Ultimately, reducing delays to surgical intervention once clinical need arises may reduce the observed disparities after TKA.

### Strengths and Limitations

Our study has several limitations. This study population was limited to women with Medicare FFS coverage; therefore, findings may not be generalizable to managed-care beneficiaries, men, or younger populations. The number of younger adults receiving TKA has increased over the years,^51^ yet disparities in the use of TKA and outcomes among adults younger than 65 years follow distinct trends from older adult populations.^52,53^ However, older women still remain the demographic group most likely to receive TKA,^54^ and given women’s longevity and the likelihood of living alone in older age,^55^ the examination of PF in this large cohort of aging women remains highly valuable. The findings for the Hispanic group should be interpreted with caution because of the large variability in PF, likely associated with the smaller sample size as well as the probable heterogeneity within this group. The RAND-36 is a generic, patient-reported measure and not a knee-specific or arthritis-specific tool nor a performance-based measure. Nevertheless, the PF scale of the RAND-36 provides a global view of self-perceived mobility required for daily living and has been widely used in studying outcomes after joint arthroplasty.^56^ Our sensitivity analysis using censoring weights demonstrated that our main findings regarding post-TKA PF are likely biased toward the null because of loss to follow-up and may in fact be an underestimation of the true disparities between racial/ethnic groups. Finally, health policy changes subsequent to the timing of the TKA procedure dates for this study may have been associated with health care use and outcomes, particularly with respect to expanded access for adults prior to or approaching Medicare eligibility. Although it is beyond the scope of this study, these findings may serve as an essential baseline for evaluating the association of the Patient Protection and Affordable Care Act and other policy changes with racial/ethnic differences in treatments and outcomes after TKA.

Balancing these limitations were several key strengths. The Medicare-WHI linked data allowed an investigation of PF from a large, diverse population of women throughout the United States using prospectively collected individual-level measurements that would not be available from medical Medicare claims data alone. Patterns in PF were examined over an extended time period, including the preoperative decade, which likely included several years prior to the first encounter with specialist care.

## Conclusions

In this study, black women had poorer PF after TKA compared with white women. The disparity in post-TKA PF was associated with disparities in PF preoperatively. Efforts to reduce the racial/ethnic gap in postoperative function should be aimed at maintaining functional mobility among black women with arthritis and at reducing delays to surgery once need arises. Investigation into the determinants of delayed use of TKA among black women warrants further attention. Additional research is also merited to understand the greater diversity in experiences and TKA outcomes among Hispanic women. Finally, it is possible that as surgical techniques and prostheses, clinical practice guidelines, and health care policies change, disparities in PF may also change, supporting the importance of continued examination of outcomes by race/ethnicity going forward.

## Notes

Supplement. **eTable 1.** Mean Differences in Physical Function Scores at Selected Time Points and Generalized Estimating Equation Parameter Estimates for Physical Function Scores of Women Post-TKA From the Women’s Health Initiative, 1993-2017, n = 7,987 **eTable 2.** Comparison of Demographic and Health Characteristics of Participants With Complete and Missing Physical Function Data **eFigure 1.** Probability of Experiencing Difficulty With Activity in the Decade Prior to TKA, by Race/Ethnicity **eFigure 2.** Preoperative Physical Function for the Decade Prior to TKA by Race/Ethnicity, With Stratification by Socioeconomic Status
