# Supplementary material for: Racial/Ethnic Disparities in Physical Function Before and After Total Knee Arthroplasty Among Women in the United States
Source: JAMA Netw Open. 2020 May 15;3(5):e204937. doi: 10.1001/jamanetworkopen.2020.4937 (PMC7229524; doi:10.1001/jamanetworkopen.2020.4937)
Supplement: Supplement. — eTable 1. Mean Differences in Physical Function Scores at Selected Time Points and Generalized Estimating Equation Parameter Estimates for Physical Function Scores of Women Post-TKA From the Women’s Health Initiative, 1993-2017, n = 7,987 eTable 2. Comparison of Demographic and Health Characteristics of Participants With Complete and Missing Physical Function Data eFigure 1. Probability of Experiencing Difficulty With Activity in the Decade Prior to TKA, by Race/Ethnicity eFigure 2. Preoperative Physical Function for the Decade Prior to TKA by Race/Ethnicity, With Stratification by Socioeconomic Status [file jamanetwopen-3-e204937-s001.pdf]

Supplementary Online Content

Cavanaugh AM, Rauh MJ, Thompson CA, et al. Racial/ethnic disparities in physical function before and after total knee arthroplasty among women in the United States. *JAMA Netw Open.* 2020;3(5):e204937. doi:10.1001/jamanetworkopen.2020.4937

eTable 1. Mean Differences in Physical Function Scores at Selected Time Points and Generalized Estimating Equation Parameter Estimates for Physical Function Scores of Women Post-TKA From the Women's Health Initiative, 1993-2017, n=7,987
eTable 2. Comparison of Demographic and Health Characteristics of Participants With Complete and Missing Physical Function Data
eFigure 1. Probability of Experiencing Difficulty With Activity in the Decade Prior to TKA, by Race/Ethnicity
eFigure 2. Preoperative Physical Function for the Decade Prior to TKA by Race/Ethnicity, With Stratification by Socioeconomic Status

This supplementary material has been provided by the authors to give readers additional information about their work.

© 2020 Cavanaugh AM et al. *JAMA Network Open.*

eTable 1. Mean Differences in Physical Function Scores at Selected Time Points and Generalized Estimating Equation Parameter Estimates for Physical Function Scores of Women Post-TKA From the Women's Health Initiative, 1993-2017, n=7,987[a]

	Physical Function Scores					
	**Unadjusted** Mean difference (95% CI)		**Adjusted for age** Mean Difference (95% CI)		**Adjusted for age and pre-TKA function** Mean Difference (95% CI)	
1 year from TKA						
Black	-6.3	(-10.8 to -3.4)	-7.8	(-10.8 to -4.9)	-3.0	(-5.3 to -0.7)
Hispanic	-1.8	(-7.4 to 3.8)	-2.6	(-8.0 to 2.7)	-1.7	(-6.0 to 2.6)
White	0	(ref)	0	(ref)	0	(ref)
2 years from TKA						
Black	-5.8	(-8.5 to -3.2)	-7.3	(-10.0 to -4.7)	-2.5	(-4.5 to -0.4)
Hispanic	-1.4	(-6.6 to 3.7)	-2.3	(-7.3 to 2.6)	-1.3	(-5.2 to 2.6)
White	0	(ref)	0	(ref)	0	(ref)
5 years from TKA						
Black	-4.3	(-6.7 to -1.9)	-5.8	(-8.2 to -3.5)	-0.9	(-2.7 to 0.9)
Hispanic	-0.3	(-5.2 to 4.6)	-1.3	(-6.0 to 3.3)	-0.2	(-3.9 to 3.6)
White	0	(ref)	0	(ref)	0	(ref)
10 years from TKA						
Black	-1.9	(-5.8 to 2.0)	-3.3	(-7.1 to 0.4)	1.7	(-1.6 to 4.9)
Hispanic	1.6	(-5.6 to 8.8)	0.3	(-6.7 to 7.4)	1.7	(-4.8 to 8.2)
White	0	(ref)	0	(ref)	0	(ref)

	**Unadjusted**			**Adjusted for age**			**Adjusted for age and pre-TKA function**		
	Estimate	Standard error	p-value	Estimate	Standard error	p-value	Estimate	Standard error	p-value
Intercept	61.68	0.68	<.001	136.32	3.55	<.001	66.22	3.04	<.001
Race/Ethnicity									
Black	-6.80	1.67	<.001	-8.32	1.68	<.001	-3.49	1.34	<.01
Hispanic	-2.21	3.12	.48	-2.99	3.00	.32	-2.05	2.46	.41
White	0	(ref)		0	(ref)		0	(ref)	
Time from TKA (years)	0.38	0.38	.31	0.29	0.37	.43	0.31	0.37	.38
Quadratic time	-0.25	0.07	<.001	-0.25	0.07	<.001	-0.25	0.07	<.001
Cubic time	0.01	0.00	.02	0.01	0.00	.02	0.01	0.00	.02
Interaction with time									
Black	0.49	0.27	.07	0.50	0.27	.06	0.52	0.24	.03
Hispanic	0.38	0.46	.41	0.33	0.46	.47	0.37	0.44	.40
White	0	(ref)		0	(ref)		0	(ref)	
Age	-	-	-	-1.00	0.05	<.001	-0.78	0.04	<.001
Pre-TKA Physical Function	-	-	-	-	-	-	0.86	0.01	<.001

[a]Physical function scores correspond to RAND-36 physical function scale
Abbreviations: TKA, total knee arthroplasty; CI, confidence interval

© 2020 Cavanaugh AM et al. *JAMA Network Open.*

eTable 2. Comparison of Demographic and Health Characteristics of Participants With Complete and Missing Physical Function Data

Characteristic	Women with Post-TKA PF scores[a]	Women without Post-TKA PF scores[b]	p-value
Race			<.001
Black/African American	410 (5.1)	212 (9.0)	
Hispanic/Latina	97 (1.2)	78 (3.3)	
White	7,465 (93.6)	2,063 (87.7)	
Age at surgery, *mean (SD)*	74 (70-78)	75 (71-79)	<.001
Region, *n (%)*			<.001
Midwest	1,780 (22.3)	425 (18.1)	
Northeast	2,292 (28.8)	804 (34.2)	
South	2,521 (31.6)	702 (29.8)	
West	1,379 (17.3)	422 (17.9)	
Marital Status, *n (% yes)*	5,295 (66.4)	1,482 (63.1)	<.01
Live Alone, n (% yes)	2,190 (29.6)	674 (31.8)	.05
BMI, *n (%)*			<.001
Underweight/Normal weight: <25 kg/m^2	1,486 (18.8)	379 (16.3)	
Overweight: 25-<30 kg/m^2	2,778 (35.1)	760 (32.7)	
Obese I: 30- <35 kg/m^2	2.099 (26.6)	630 (27.1)	
Obese II/II: ≥35 kg/m^2	1,544 (19.5)	555 (23.9)	
Multimorbidity Score (CMS-HCC), *median (IQR)*	0.62 (0.43-0.92)	0.67 (0.43-1.01)	<.001
Moderate-to-Strenuous Physical Activity, *n (%)*			<.001
None	3,084 (38.7)	1,087 (46.5)	
1-<150 minutes/week	2,675 (33.6)	723 (31.0)	
>150 minutes/week	2,210 (27.7)	525 (22.5)	
Depressive Symptoms, n (% yes)	697 (8.8)	309 (13.3)	<.001
Educational Level, *n (%)*			<.001
Less than high school	212 (2.7)	116 (5.0)	
High school	1,310 (16.5)	508 (21.7)	
Some college	2,928 (36.9)	936 (39.9)	
College graduate	3,490 (43.9)	785 (33.5)	
Family Income, *n (%)*			<.001
<$20,000	730 (9.7)	375 (17.3)	
$20,000-<$50,000	34,448 (45.7)	1,019 (47.0)	
≥$50,000	3,370 (44.7)	775 (35.7)	
Neighborhood SES, *mean (SD)*	77.8 (73.0-81.7)	77.2 (72.1-81.1)	<.001

[a] Included in physical function generalized estimating equation models.
[b] Excluded from physical function generalized estimating equation models due to incomplete data

© 2020 Cavanaugh AM et al. *JAMA Network Open.*

eFigure1. Probability of experiencing difficulty with activity in the decade prior to TKA, by race ethnicity

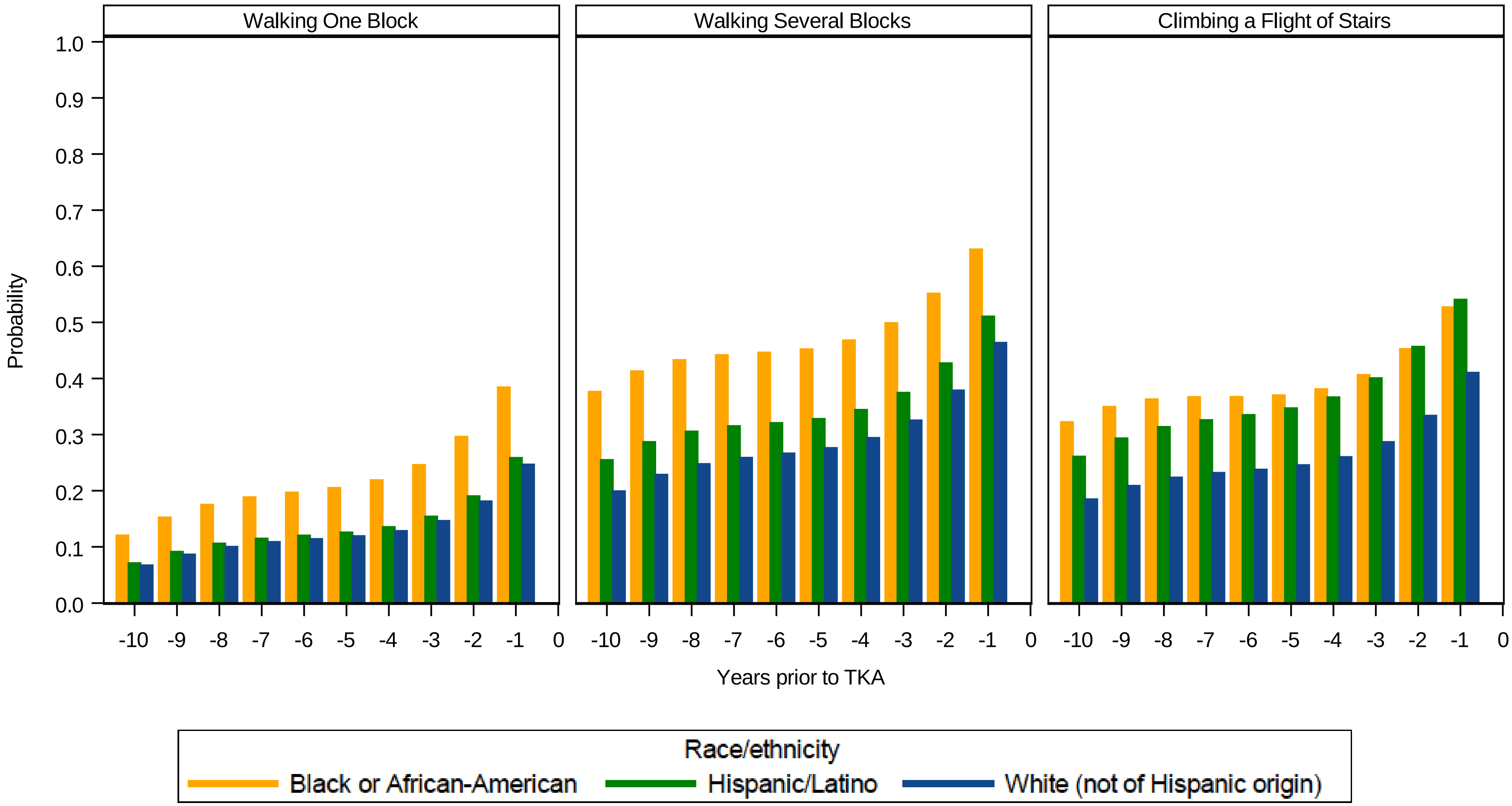

Predicted probability determined through generalized estimating equation models fit for age 74 at time of TKA

© 2020 Cavanaugh AM et al. *JAMA Network Open.*

eFigure 2.Pre-operative physical function for the decade prior to TKA by race/ethnicity, with stratification by socioeconomic status.

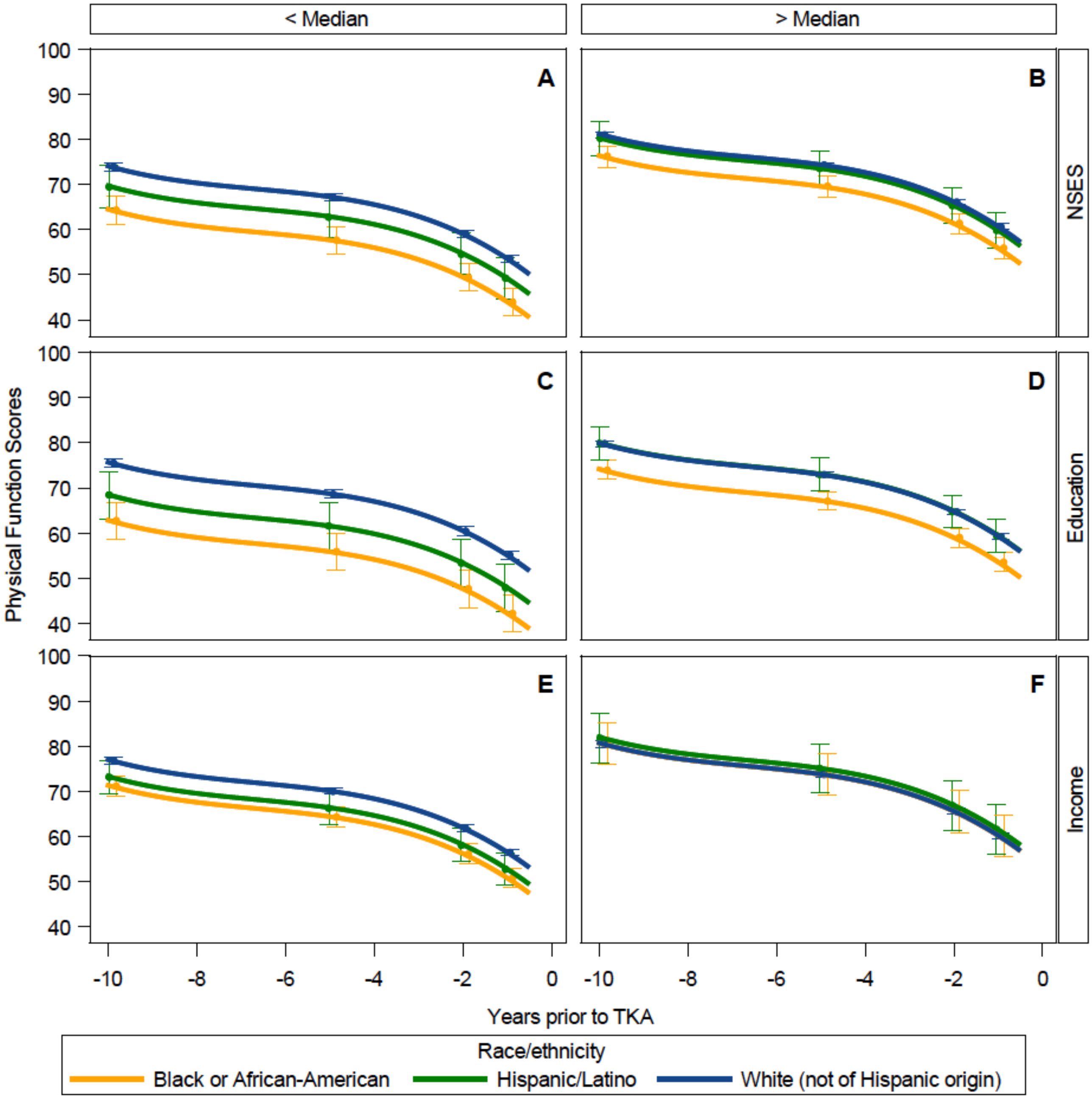

Values are predicted marginal means from GEE models fit for age 74 at time of TKA.
A) education of high school completion/GED or lesser attainment B) educational attainment of some college
C) annual family income < $35,000 D) annual family income $35,000 or greater
E) neighborhood socioeconomic status (NSES) <77.68 F) NSES 77.68 or greater

© 2020 Cavanaugh AM et al. *JAMA Network Open.*
